# Quantitative proteomic analyses of CD4^+^ and CD8^+^ T cells reveal differentially expressed proteins in multiple sclerosis patients and healthy controls

**DOI:** 10.1186/s12014-019-9241-5

**Published:** 2019-05-08

**Authors:** Tone Berge, Anna Eriksson, Ina Skaara Brorson, Einar August Høgestøl, Pål Berg-Hansen, Anne Døskeland, Olav Mjaavatten, Steffan Daniel Bos, Hanne F. Harbo, Frode Berven

**Affiliations:** 1 Department of Mechanical, Electronics and Chemical Engineering, Faculty of Technology, Art and Design, Oslo Met – Oslo Metropolitan University, Postboks 4, St. Olavs Plass, 0130 Oslo, Norway; 2 Neuroscience Research Unit, Oslo University Hospital, Rikshospitalet, Domus Medica 4, Nydalen, Postboks 4950, 0424 Oslo, Norway; 3 Department of Research, Innovation and Education, Oslo University Hospital, Oslo, Norway; 4 Institute of Clinical Medicine, University of Oslo, Oslo, Norway; 5 Department of Neurology, Oslo University Hospital, Ullevål, Postboks 4950, 0424 Nydalen, Oslo, Norway; 6 Proteomics Unit at University of Bergen (PROBE), Department of Biomedicine, University of Bergen, Postboks 7804, 5020 Bergen, Norway

**Keywords:** Multiple sclerosis, T cells, Mass spectrometry, SNPs, Autoimmunity, Proteomics

## Abstract

**Background:**

Multiple sclerosis (MS) is an autoimmune, neuroinflammatory disease, with an unclear etiology. However, T cells play a central role in the pathogenesis by crossing the blood–brain-barrier, leading to inflammation of the central nervous system and demyelination of the protective sheath surrounding the nerve fibers. MS has a complex inheritance pattern, and several studies indicate that gene interactions with environmental factors contribute to disease onset.

**Methods:**

In the current study, we evaluated T cell dysregulation at the protein level using electrospray liquid chromatography–tandem mass spectrometry to get novel insights into immune-cell processes in MS. We have analyzed the proteomic profiles of CD4^+^ and CD8^+^ T cells purified from whole blood from 13 newly diagnosed, treatment-naive female patients with relapsing–remitting MS and 14 age- and sex-matched healthy controls.

**Results:**

An overall higher protein abundance was observed in both CD4^+^ and CD8^+^ T cells from MS patients when compared to healthy controls. The differentially expressed proteins were enriched for T-cell specific activation pathways, especially CTLA4 and CD28 signaling in CD4^+^ T cells. When selectively analyzing proteins expressed from the genes most proximal to > 200 non-HLA MS susceptibility polymorphisms, we observed differential expression of eight proteins in T cells between MS patients and healthy controls, and there was a correlation between the genotype at three MS genetic risk loci and protein expressed from proximal genes.

**Conclusion:**

Our study provides evidence for proteomic differences in T cells from relapsing–remitting MS patients compared to healthy controls and also identifies dysregulation of proteins encoded from MS susceptibility genes.

**Electronic supplementary material:**

The online version of this article (10.1186/s12014-019-9241-5) contains supplementary material, which is available to authorized users.

## Background

Multiple sclerosis (MS) typically affects young adults and is the most common non-traumatic cause of neurological impairment. It affects around 2.5 million individuals worldwide leading to both physical and cognitive deficits [1]. MS is a chronic inflammatory, demyelinating disorder of the central nervous system (CNS) where lymphocyte-mediated inflammation causes demyelination and axonal degeneration. The underlying pathogenesis remains partly unclear, but T lymphocytes, both CD4^+^ and CD8^+^ T cells, have long been considered to play pivotal roles in MS pathogenesis [2, 3]. Also, the genetic architecture of MS susceptibility, emerging from genome-wide association studies, indicates an important role for the adaptive immune system, in particular T cells for MS-disease onset [4, 5].

Studies of MS etiology in monozygotic twins and recurrence risk in siblings indicate that MS has a complex inheritance pattern [6]. Furthermore, parent-of-origin effects affect inheritance of MS in rodents, and several studies indicate that gene-environment interactions contribute to MS development. Altogether, this suggests that also epigenetic mechanisms play a role in MS etiology [7]. Both genome-wide studies on epigenetic modifications, such as DNA methylation, as well as transcriptomic analyses in immune cells have been conducted in order to investigate the potential dysregulation of immune cells in MS. Epigenetic profiling in peripheral blood mononuclear cells and in immune cell subtypes, i.e. CD4^+^ and CD8^+^ T cells, suggests global differences in DNA methylation between MS patients and healthy controls [8–12]. Of note, a few single genes displayed significant differential DNA methylation levels between MS patients and healthy controls, but no overlap, except for in the HLA-DRB1 locus [12, 13], was observed between the different studies [7]. Microarray analyses of blood from MS patients and healthy controls indicate dysregulation of T cell pathways during MS pathogenesis [14, 15]. Recent candidate-gene approaches have profiled transcriptional changes in T cells from MS cases and healthy controls, and identified dysregulation of several genes, e.g. *MIR*-*21* and corresponding target genes [16] and *THEMIS* [17]. However, the correlation between mRNA and protein copy numbers varies widely [18, 19]. Therefore, performing quantitative high-resolution mass spectrometry-based proteomics gives a unique opportunity for system-wide studies at the protein level.

Since the 1970′ies, HLA-DRB1*15:01 has been established as the major genetic risk factor in MS [6]. Recent genome-wide screenings have however identified more than 200 non-HLA single nucleotide polymorphisms (SNPs) associated with MS risk [4, 5, 20]. The majority of the non-HLA MS associated SNPs are non-coding, and an enrichment of these variants is observed in regulatory regions of DNA (DNase hypersensitive sites) in immune cells from the adaptive arm of the immune system, i.e. B and T cells [21]. In addition, given the widespread presence of expression quantitative trait loci (eQTLs) in the genome [22], it is likely that a number of MS-associated SNPs or SNPs inherited together with the MS-associated SNPs might act as eQTLs in immune cells. Indeed, a recent study identified 35 significant eQTLs from 110 non-HLA MS-associated SNPs in peripheral blood mononuclear cells from MS patients [23]. However, whether these expression differences at the transcriptomic levels also persists to the protein level is currently unknown.

The overall objective for this project is to evaluate immune dysregulation at the protein level in MS using liquid chromatography combined with mass spectrometry. We analyzed the proteomic profile of purified immune-cell subsets, i.e. CD4^+^ and CD8^+^ T cells, from genotyped relapsing–remitting MS (RRMS) patients and healthy controls, which allows us to disentangle potential cell-subtype specific differences that could not be detected in a heterogeneous cell material, permitting a comprehensive understanding of disease mechanisms of MS. Correlating protein expression with genotypes of MS-associated SNPs allowed for identification of protein expression quantitative trait loci (pQTLs).

## Methods

### MS patients and healthy controls

Samples from 13 untreated, female Norwegian MS patients with RRMS and 14 age-matched, female Norwegian healthy controls were included (see Table 1 for demographic, clinical and biochemical information). For two of the patients, the EDSS score was assessed by inspection of their medical journals. All patients and healthy controls were self-declared of Nordic ancestry. Patients were recruited from the MS out-patient clinic at the Oslo University Hospital, Oslo, Norway and the healthy controls among hospital employees. All MS patients fulfilled the updated McDonald criteria for MS [24], did not have an ongoing infection and had not experienced a relapse or received steroids in the 3 months prior to enrollment. The diagnosis was set less than 1 year prior to inclusion in the study. The healthy controls did report to have no MS in near family.

**Table 1:** Characteristics of individual MS patients and summaries of patients and healthy controls

Patient	Age category^a^	Years since first MS symtoms	EDSS	MSSS	OCB	MRI lesion categories^b^	Contrast lesions MRI	Symptoms at onset	Family history of MS
MS1	3	6	2.5	7.1	Yes	3	Yes	Visual	No
MS2	1	4	1	2.44	Yes	2	Yes	Brainstem	Yes
MS3	6	7	3	7.93	Yes	1	No	Visual	Yes
MS4	1	0.75	1.5	4.3	Yes	1	Yes	Sensory	No
MS5	1	15	3.5	8.64	Yes	1	No	Sensory	No
MS6	4	0.75	2	5.87	Yes	3	Yes	Brainstem	No
MS7	2	0.5	1	2.44	Yes	3	No	Sensory	No
MS8	4	2	1	2.44	Yes	3	Yes	Visual	Yes
MS9	5	3	2.5	7.08	No	3	Yes	Sensory, bladder/bowel	No
MS10	1	0.75	3	7.93	Yes	1	Yes	Pyramidal	Yes
MS11	6	19	1.5	4.3	Yes	1	No	Sensory	No
MS12	5	14	2.5	7.08	Yes	2	No	Visual	No
MS13	1	1	1.5	4.3	Yes	2	Yes	Sensory	No
*Summarized*									
Patients mean or median* (range)	37.2 (25–52)	5.7 (0.75–19)	2 (1–3.5)*	5.5 (2.4–8.6)	N/A	2*	N/A	N/A	N/A
Healthy controls mean (range)	32.6 (23–47)	N/A	N/A	N/A	N/A	N/A	N/A	N/A	N/A

The table includes data for each individual MS patient at inclusion, from the left: patient identity number; ^a^age category; number of years since first MS symptoms; EDSS; MSSS; presence of OCB in the cerebrospinal fluid; ^b^MRI lesion categories; presence of contrast enhancing lesions (MRI); symptoms at onset and family history of MS. Below follows summary statistics with mean (range) for age category, years since first symptoms and MSSS and median (range) labelled with * for EDSS and MRI lesion categories

*EDSS* expanded disability status scale, *MSSS* MS severity score, *OCB* oligoclonal bands, *MRI* magnetic resonance imaging, *N/A* not applicable

^a^Age category: 1 = 25–29 years; 2 = 30–34 years; 3 = 35–39 years; 4 = 40–44 years; 5 = 45–49 years; 6 = 50–54 years

^b^MRI lesion categories:: 1 = 0–10 lesions; 2 = 10–20 lesions; 3 = more than 20 lesions

### DNA isolation and genotyping

DNA was purified from blood (DNeasy Blood & Tissues Kit, Qiagen, Redwood City, CA, USA). Samples were genotyped with the Human Omni Express BeadChip (Illumina, San Diego, CA, USA).

### Isolation of human CD4^+^ and CD8^+^ T cells, sample preparation and protein digestion

Peripheral blood mononuclear cells were isolated from whole blood by Lymphoprep (Axis Shield, Dundee, Scotland), before positive selection of CD8^+^ T cells (EasySep™ Human CD8^+^ Selection Kit, STEMCELL Technologies, Vancouver, Canada) followed by negative selection of CD4^+^ T cells (EasySep™ Human CD4^+^ T cell Isolation kit, STEMCELL Technologies). Cells that achieved cell purity of more than 95% as measured by flow cytometry (Attune Acoustic Focusing Flow Cytometer, Life Technologies, Carlsbad, CA, USA) were included in the study. Two CD8^+^ T cell samples from MS patients did not reach 95% cell purity and were excluded from the analyses. Antibodies used for flow cytometry analyses were fluorescein isothiocyanate-conjugated mouse anti-human CD4 (clone RTF-4 g, Southern Biotech, Birmingham, AL, USA), mouse anti-human CD8 (clone HIT8a, BD biosciences, San Jose, CA, USA) and mouse IgG1 isotype control (15H6, Southern Biotech).

### Sample preparation and protein digestion

The pellet of 1 × 10^6^ cells from each sample was kept until use at − 80 °C. The pellets were then solubilized in 100 μl 0.1 M Tris–HCl pH 7.6 containing 4% SDS and homogenized at room temperature by sonication 3–4 times at 30% amplitude for 30 s with an ultrasonic processor with thumb-petuated pulser (Vibra-cell VC130 PB from Sonics and Materials Inc., Newton, CT, USA). After centrifugation for 10 min at 16,200 × *g*, supernatants were collected. Protein concentration in samples was measured by Pierce BCA protein assay (Thermo Fisher Scientific, Rockford, IL, USA) and the absorbance values at 562 nm were read on Multiskan FC 3.1 ELISA reader (Thermo Fisher Scientific). To 40 μl supernatant corresponding to about 10 μg protein, 4 μl 1 M DTT was added for reduction and incubated at 95 °C for 5 min. After cooling, SDS removal by dilution with urea and cysteine alkylation, digestion of proteins were accomplished using the filter aided sample preparation (FASP) protocol [25]. On the Microcon^R^-30 centrifugal filters (Merck Millipore Ltd, Ireland), proteins were digested with a protein-to-trypsin ratio of 50:1 (sequencing grade-modified trypsin from Promega, GmbH, Mannheim, Germany) [26]. After incubation overnight at 37 °C, tryptic peptides were collected by washing the filter three times with 50 mM ammonium bicarbonate pH 8.5, and with 0.5 M NaCl, each step followed by centrifugation at 11,000 × *g* [25]. Sample cleanup was performed using a reverse-phase Oasis^R^ HLB μElution Plate 30 μm (2-mg HLB sorbent, Waters, Milford, MA) [27]. After lyophilization, the dried peptides were suspended in 12 μl of 0.1% formic acid containing 2% acetonitrile. 2 μl were used for protein quantification based on absorbance at 280 nm using a NanoDrop spectrophotometer (Thermo Fisher Scientific). The sample volume was adjusted to 1 μg/μl and approximately 1 μg of the mixture was analyzed with mass spectrometry.

### Liquid chromatography–mass spectrometry/mass spectrometry analysis

The peptides were analyzed by electrospray liquid chromatography–tandem mass spectrometry (LC–MS/MS) using a linear ion trap–orbitrap instrument (Orbitrap Elite, Thermo Fisher Scientific). The LC run length of 3 h was performed on a 50 cm analytical column (Acclaim PepMap 100, 50 cm × 75 µm ID nanoViper column, packed with 3 µm C18 beads (Thermo Fisher Scientific)). Peptides were loaded and desalted on a pre-column (Acclaim PepMap 100, 2 cm × 75 µm ID nanoViper column, packed with 3 µm C18 beads (Thermo Fisher Scientific)) with 0.1% (v/v) trifluoroacetic acid, and eluted with a gradient composition as follows: 5% B during trapping (5 min) followed by 5–7% B over 1 min, 7–32% B for the next 129 min, 32–40% B over 10 min, and 40–90% B over 5 min. Elution of very hydrophobic peptides and conditioning of the column were performed during 20 min isocratic elution with 90% B and 20 min isocratic elution with 5% B respectively. Mobile phases A and B with 0.1% formic acid (vol/vol) in water and 100% acetonitrile respectively, and the flow rate was of 270 nl per min. A full scan in the mass area of 300–2000 Da was performed in the Orbitrap. For each full scan performed at a resolution of 240,000, the 12 most intense ions were selected for collision induced dissociation (CID). The settings of the CID were as following: threshold for ion selection was 3000 counts, the target of ions used for CID was 1e4, activation time was 10 ms, isolation window was 2 Da, and normalized collision energy was 35 eV.

### Mass spectrometry data analysis

MS raw files were analyzed by the MaxQuant software [28] (version 1.5.6.0), and peak lists were searched against the human SwissProt FASTA database (version May 2017), and a common contaminants database by the Andromeda search engine. As variable modification, methionine oxidation was used and as fixed modification cysteine carbamidomethylation was used. False discovery rate was set to 0.01 for proteins and peptides (minimum length of six amino acids) and was determined by searching a reverse database. Trypsin was set as digestion protease, and a maximum of two missed cleavages were allowed in the database search. Peptide identification was performed with an allowed MS mass deviation tolerance of 20 ppm, and MS/MS fragment ions could deviate by up to 0.5 Da. For accurate intensity-based label-free quantification in MaxQuant [MaxLFQ [29]], the type of label was “1″ for LFQ with a minimum ratio count of “2″. For matching between runs, the retention time alignment window was set to 20 min and the match time window was 0.7 min.

### Statistical analyses

The statistical significance between comparisons was evaluated using a two-tailed Student *t* test, p < 0.05 was considered significant. The equality of variances of patient and control distributions was assessed with an F-test. Consequently, a Student *t* test with unequal variances was used when the F-test was significant (p < 0.05) and with equal variances otherwise. Area under the ROC curve (AUC) analyses of all significantly expressed proteins (p < 0.05) was calculated using GraphPad Prism 6 (La Jolla, CA, USA). Individual scatter plots of selected proteins (Figs. 4, 5) was created using GraphPad Prism 6. For the genotype-wise comparisons, a Students unpaired t-test with equal variances was performed when the data were normally distributed, if not, the non-parametric Mann *U* Whitney test was performed (GraphPad Prism 6).

### Data processing, principal component and hierarchical clustering analyses

Proteins identified as “only identified by site”, “reverse” or “potential contaminant” by Max Quant were removed from further analyses. In Perseus (Perseus Software, version 1.6.0.7), the normalized LFQ intensities from Max Quant were log2 transformed and the normal distributions were controlled using histogram function for each individual. Proteins with at least 70 percentage valid values in each group (healthy control and MS) were analyzed. Further, hierarchical clustering was performed using Z-scores created by default settings in Perseus. A principal component analysis (PCA) plot was generated using protein intensities as variables, with the missing protein intensity values imputed from the normal distribution using default settings in Perseus.

### Ingenuity pathway analyses

QIAGEN’s Ingenuity^®^ pathway Analysis (IPA^®^, QIAGEN, version 44691306 date; 2018-06-15, build version: 481437M date; 2018-08-25) was used for functional interpretation of significantly regulated proteins. The default settings were used, except only the following confidence, species and tissues and cells were permitted: “only experimentally observed” (confidence), “only mammals” (species) and “only T cells” (primary and cell-lines (tissues and cells)). A Benjamin-Hochberg (B-H) multiple testing correction was used, where a −log(B-H p-value) of 1.3 was considered as significant.

## Results

### Differential protein expression is observed in T cells between MS patients and healthy control

**Fig. 1:**
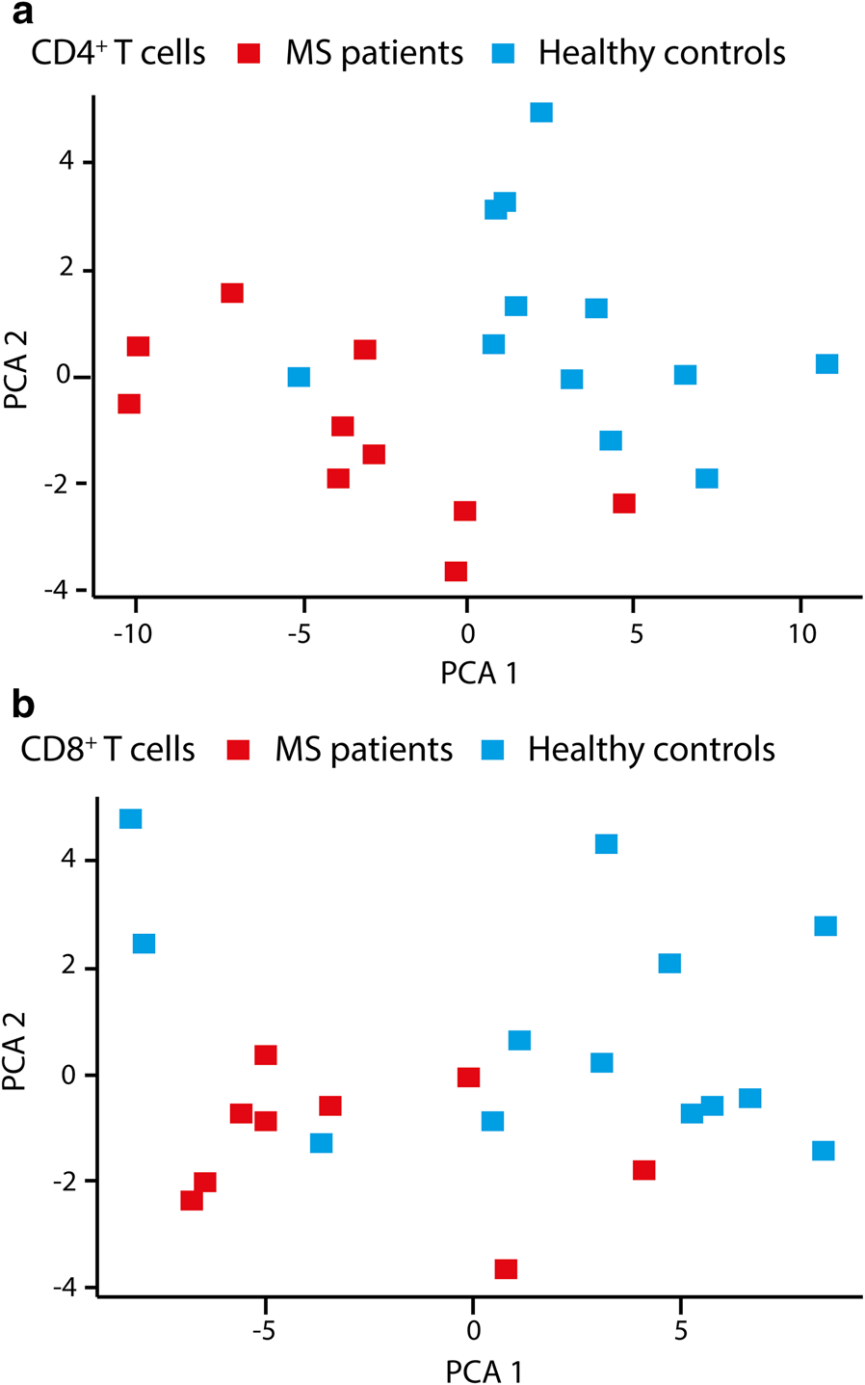
Principal component analyses (PCA) of differentially expressed proteins. PCA of proteins significantly different (p < 0.05) in **a** CD4^+^ and **b** CD8^+^ T cells from MS cases (red) compared to healthy controls (blue)

In this study, we monitored the difference in the proteomic profiles in T cells, i.e. CD4^+^ and CD8^+^ T cells, between RRMS patients (n = 13) and healthy controls (n = 14) in a label-free manner. We were able to identify and quantify 2031 and 2259 proteins in CD4^+^ and CD8^+^ T cells, respectively. In CD4^+^ T cells, 228 proteins were differentially expressed (p < 0.05) between MS cases and healthy controls (listed in Additional file 1: Table S1), whereas 195 proteins were differentially expressed between the two groups in CD8^+^ T cells (listed in Additional file 2: Table S2). Of the differentially expressed proteins, 74% in CD4^+^ T cells and 64% in CD8^+^ T cells were more abundant in samples from MS patients compared to healthy controls. The separation of MS versus healthy controls based on these proteins is shown in the principal component analyses (PCA) plot in Fig. 1, where the first component captures 55% (CD4^+^) and 62% (CD8^+^) of the variance, whereas the second component captures 11% (CD4^+^) and 9% (CD8^+^). Of the differentially expressed proteins, 26 overlapped between CD4^+^ and CD8^+^ T cells.

### Ingenuity pathway analyses of differentially expressed proteins

To increase the chance of extracting the true candidate proteins differentially expressed between MS cases and healthy controls with a potential impact on cell function, a more stringent filter for selection was applied. By selecting proteins that fulfilled two of the three following criteria within the group of significantly differential expressed proteins (p < 0.05): (1) p-value cut-off of p < 0.01; (2) area under the curve (AUC) > 0.8 and (3) log2 fold change > [0.2], we created a top-hit list of differentially expressed proteins. Out of the 228 and 195 proteins listed in Additional file 1: Table S1 and Additional file 2: Table S2 from CD4^+^ and CD8^+^ T cells, respectively, we ended up with a shorter list of 90 and 61 proteins (Tables 2, 3), where five proteins expressed from the *TOMM70A*, *ACP1*, *AGL*, *ATP2A2* and *TPM4* genes appeared in both top-hit lists.

**Table 2:** Top-hit list of differentially expressed proteins in CD4^+^ T cells

Accession	Protein identity	Gene names	p-value	FC MS versus HC (log2)	Median intensity MS (log2)	MS SD	Median intensity HC (log2)	HC SD	% seq cov	# pep	AUC
Q5JSL3	Dedicator of cytokinesis protein 11	DOCK11	4.69E−05	0.27405	22.73205	0.14968	22.458	0.11384	13	21	0.98
Q03252	Lamin-B2	LMNB2	0.000203	0.2023	26.23395	0.10367	26.03165	0.1219	58.1	42	0.94
Q14978	Nucleolar and coiled-body phosphoprotein 1	NOLC1	0.000306	0.67815	21.4053	0.26237	20.72715	0.36787	16.2	9	0.92
Q2M2I8; Q9NSY1	AP2-associated protein kinase 1	AAK1	0.000457	0.22605	23.1178	0.11404	22.89175	0.12897	33	20	0.92
Q13148	TAR DNA-binding protein 43	TARDBP	0.000642	0.29	23.3943	0.12816	23.1043	0.14754	39.4	11	0.89
P20963	T-cell surface glycoprotein CD3 zeta chain	CD247	0.000907	0.19535	23.48275	0.0965	23.2874	0.18125	60.4	11	0.88
P49959	Double-strand break repair protein MRE11A	MRE11A	0.001405	0.1957	21.44665	0.17074	21.25095	0.15881	21.6	11	0.88
P06239	Tyrosine-protein kinase Lck	LCK	0.001598	0.2009	24.642	0.12459	24.4411	0.13158	49.7	18	0.85
Q9NR56; Q5VZF2; Q9NUK0	Muscleblind-like protein 1	MBNL1	0.001651	0.3464	22.0867	0.19817	21.7403	0.24361	21.6	8	0.87
P35573	Glycogen debranching enzyme; 4-alpha-glucanotransferase; amylo-alpha-1,6-glucosidase	AGL	0.00177	0.32245	21.79335	0.29915	21.4709	0.18837	18.1	18	0.87
P18085	ADP-ribosylation factor 4	ARF4	0.00199	− 0.29765	21.6712	0.17375	21.96885	0.14457	64.4	10	0.86
O75131; Q96FN4; Q8IYJ1; Q9HCH3; Q9UBL6	Copine-3	CPNE3	0.002255	0.1118	23.9288	0.09682	23.817	0.07363	46.7	19	0.88
P27824	Calnexin	CANX	0.002331	− 0.2029	24.6288	0.09381	24.8317	0.13864	37.7	22	0.85
Q49A26	Putative oxidoreductase GLYR1	GLYR1	0.002442	0.2299	22.8002	0.15088	22.5703	0.13549	40	14	0.88
P12694	2-oxoisovalerate dehydrogenase subunit alpha, mitochondrial	BCKDHA	0.002513	0.2997	20.58005	0.14155	20.28035	0.16289	21.1	6	0.89
P16615	Sarcoplasmic/endoplasmic reticulum calcium ATPase 2	ATP2A2	0.002577	− 0.34015	20.91155	0.24663	21.2517	0.39528	22.5	15	0.85
P31146; REV__Q02818	Coronin-1A	CORO1A	0.002667	0.196	28.77805	0.04311	28.58205	0.14531	63.8	33	0.91
P29401	Transketolase	TKT	0.002709	0.18195	27.0961	0.16375	26.91415	0.08497	68.9	38	0.86
Q00610; P53675	Clathrin heavy chain 1	CLTC	0.00312	− 0.10695	26.3723	0.05858	26.47925	0.08019	58.7	80	0.83
P19971	Thymidine phosphorylase	TYMP	0.003318	− 0.6095	21.51775	0.63532	22.12725	0.52772	51	16	0.85
Q16401	26S proteasome non-ATPase regulatory subunit 5	PSMD5	0.003478	0.12765	23.7053	0.09891	23.57765	0.13094	58.9	21	0.86
Q15084	Protein disulfide-isomerase A6	PDIA6	0.003546	− 0.3043	23.5948	0.25739	23.8991	0.17192	45.9	13	0.86
P07237	Protein disulfide-isomerase	P4HB	0.003888	− 0.1857	25.1359	0.14266	25.3216	0.09151	56.1	27	0.85
O43665	Regulator of G-protein signaling 10	RGS10	0.003925	0.2594	23.5918	0.213	23.3324	0.14464	60.1	12	0.85
P27986; O00459	Phosphatidylinositol 3-kinase regulatory subunit alpha	PIK3R1	0.004008	0.2604	22.56095	0.17873	22.30055	0.21783	38.3	19	0.83
Q9Y4L1	Hypoxia up-regulated protein 1	HYOU1	0.004021	− 0.1815	23.00205	0.13058	23.18355	0.13156	31.8	20	0.83
O75306	NADH dehydrogenase [ubiquinone] iron-sulfur protein 2, mitochondrial	NDUFS2	0.004057	0.13545	22.6738	0.08259	22.53835	0.13156	34.8	12	0.83
Q8WUX9	Charged multivesicular body protein 7	CHMP7	0.004115	0.23275	21.9775	0.21092	21.74475	0.18291	37.1	13	0.81
P07602	Prosaposin; Saposin-A; Saposin-B-Val; Saposin-B; Saposin-C; Saposin-D	PSAP	0.004366	− 0.19325	22.296	0.18336	22.48925	0.42157	12.6	6	0.94
O00422	Histone deacetylase complex subunit SAP18	SAP18	0.004452	0.37715	20.6193	0.18761	20.24215	0.34985	41.8	5	0.87
Q9ULA0	Aspartyl aminopeptidase	DNPEP	0.004664	0.3613	23.6397	0.17228	23.2784	0.18788	53.3	18	0.82
O43681	ATPase ASNA1	ASNA1	0.004954	− 0.11665	22.25215	0.13672	22.3688	0.11129	50.6	10	0.83
O75832	26S proteasome non-ATPase regulatory subunit 10	PSMD10	0.004963	0.21305	21.312	0.24569	21.09895	0.12837	40.3	6	0.89
P30536	Translocator protein	TSPO	0.004964	0.5376	22.44845	0.37985	21.91085	0.337	23.1	3	0.82
P24666	Low molecular weight phosphotyrosine protein phosphatase	ACP1	0.005013	0.2241	22.8028	0.19373	22.5787	0.20543	72.2	8	0.88
Q4G176	Acyl-CoA synthetase family member 3, mitochondrial	ACSF3	0.005127	0.3234	20.8339	0.32659	20.5105	0.20115	19.3	7	0.83
P35611	Alpha-adducin	ADD1	0.005201	0.17245	23.941	0.12213	23.76855	0.20616	44.9	24	0.81
P19525	Interferon-induced, double-stranded RNA-activated protein kinase	EIF2AK2	0.005211	− 0.54585	20.65625	0.47474	21.2021	0.40633	20.1	9	0.87
O75791	GRB2-related adapter protein 2	GRAP2	0.00589	0.1927	23.58335	0.07421	23.39065	0.15601	43	13	0.84
Q16666; Q6N021	Gamma-interferon-inducible protein 16	IFI16	0.006051	− 0.27745	24.51685	0.24674	24.7943	0.12775	43.4	31	0.84
Q9HAV4	Exportin-5	XPO5	0.006457	− 0.402	18.4781	0.23884	18.8801	0.22546	5.1	4	0.87
Q9NRY5	Protein FAM114A2	FAM114A2	0.006779	0.4935	19.3331	0.23485	18.8396	0.34369	15.8	4	0.86
P11177	Pyruvate dehydrogenase E1 component subunit beta, mitochondrial	PDHB	0.006838	0.2322	24.05355	0.11379	23.82135	0.12468	52.9	13	0.83
Q9NZZ3	Charged multivesicular body protein 5	CHMP5	0.006962	− 0.28845	20.37145	0.31795	20.6599	0.20311	40.6	6	0.83
P53634	Dipeptidyl peptidase 1; dipeptidyl peptidase 1 exclusion domain chain; dipeptidyl peptidase 1 heavy chain; dipeptidyl peptidase 1 light chain	CTSC	0.006992	− 0.36305	20.5409	0.54754	20.90395	0.10359	19.9	7	0.81
Q06546	GA-binding protein alpha chain	GABPA	0.006996	0.2074	21.3763	0.1983	21.1689	0.20734	28	8	0.8
P21399	Cytoplasmic aconitate hydratase	ACO1	0.008051	0.1699	21.4757	0.14153	21.3058	0.20875	20.4	11	0.82
Q9H400	Lck-interacting transmembrane adapter 1	LIME1	0.008125	0.25515	21.11	0.19997	20.85485	0.21307	46.1	7	0.81
Q02750	Dual specificity mitogen-activated protein kinase kinase 1	MAP2K1	0.00822	0.1771	23.2231	0.13291	23.046	0.1348	42.2	14	0.8
O94826	Mitochondrial import receptor subunit TOM70	TOMM70A	0.008231	0.21725	22.34995	0.15186	22.1327	0.20502	34.5	13	0.81
O75475	PC4 and SFRS1-interacting protein	PSIP1	0.008443	0.1899	22.08185	0.1504	21.9335	0.15516	45.5	21	0.8
P02776	Platelet factor 4; platelet factor 4, short form	PF4	0.008535	− 1.5035	24.86845	1.22842	24.67855	1.48716	36.6	5	0.83
Q5XKP0	Protein QIL1	QIL1	0.008552	0.31595	22.7718	0.27181	24.2753	0.34286	62.7	3	0.84
Q9UGI8	Testin	TES	0.008688	0.14215	19.94335	0.09764	19.6274	0.12853	72	31	0.8
Q86VP6; O75155	Cullin-associated NEDD8-dissociated protein 1	CAND1	0.008724	0.11355	25.3342	0.10321	25.19205	0.08176	48.9	46	0.84
Q9C0K0	B-cell lymphoma/leukemia 11B	BCL11B	0.008892	0.2434	25.65085	0.17505	25.5373	0.22495	12.8	8	0.79
P13861; P31323	cAMP-dependent protein kinase type II-alpha regulatory subunit	PRKAR2A	0.008993	0.13145	21.90015	0.12538	21.65675	0.09173	62.1	20	0.81
P07741	Adenine phosphoribosyltransferase	APRT	0.008995	0.19165	23.14455	0.1699	23.0131	0.15824	91.1	17	0.83
P23246	Splicing factor, proline- and glutamine-rich	SFPQ	0.009648	0.12175	25.8719	0.14919	25.68025	0.09657	47.9	31	0.83
P49903	Selenide, water dikinase 1	SEPHS1	0.009747	0.2257	26.39505	0.15139	26.2733	0.15099	41.6	10	0.83
P62995	Transformer-2 protein homolog beta	TRA2B	0.009757	0.17515	22.6718	0.18232	22.4461	0.12504	30.9	8	0.8
Q86XP3	ATP-dependent RNA helicase DDX42	DDX42	0.009985	0.1467	23.91205	0.20211	23.7369	0.12402	22.7	13	0.85
P13010	X-ray repair cross-complementing protein 5	XRCC5	0.01116	0.2196	22.23445	0.1468	22.08775	0.12306	71.2	48	0.82
Q15428	Splicing factor 3A subunit 2	SF3A2	0.011498	0.30175	25.0703	0.2546	25.26055	0.28193	28.7	9	0.85
P37837	Transaldolase	TALDO1	0.011683	0.26525	24.0953	0.16309	23.9199	0.1934	47.2	19	0.8
O94973	AAK1	AP2A2	0.01208	0.40715	22.87215	0.18724	22.7054	0.31295	25	16	0.82
P16150	Leukosialin	SPN	0.012636	0.41995	27.0869	0.31488	26.8673	0.23838	19.5	5	0.8
Q9Y6K5	2-5-oligoadenylate synthase 3	OAS3	0.013062	− 0.58225	24.1071	0.61142	23.9796	0.40043	26.2	21	0.8
P13598	Intercellular adhesion molecule 2	ICAM2	0.013215	− 0.33575	22.4839	0.36073	22.18215	0.13532	14.9	3	0.81
O96000	NADH dehydrogenase [ubiquinone] 1 beta subcomplex subunit 10	NDUFB10	0.013266	0.2682	27.0077	0.2602	26.74245	0.17232	43	7	0.82
P48059; Q7Z4I7	LIM and senescent cell antigen-like-containing domain protein 1	LIMS1	0.013613	− 1.11835	22.2423	1.06748	22.4974	1.04857	45.8	13	0.83
P0DOX5; P01857	Ig gamma-1 chain C region	IGHG1	0.014981	− 0.8553	21.9197	0.96324	21.51255	0.41275	28.3	9	0.8
P67936	Tropomyosin alpha-4 chain	TPM4	0.015875	− 0.39585	22.849	0.50209	22.42905	0.33951	66.1	27	0.81
Q53QZ3	Rho GTPase-activating protein 15	ARHGAP15	0.016084	0.2283	22.7616	0.11442	22.6226	0.26067	28.8	10	0.8
Q93077; Q7L7L0; P04908	Histone H2A type 1-C; histone H2A type 3; histone H2A type 1-B/E	HIST1H2AC; HIST3H2A; HIST1H2AB	0.016472	0.5318	24.6077	0.68101	24.4913	0.51829	35.4	7	0.83
Q00341	Vigilin	HDLBP	0.017653	− 0.3557	22.1316	0.25975	22.71385	0.26847	5.3	5	0.8
Q9Y3C4	EKC/KEOPS complex subunit TPRKB	TPRKB	0.01784	0.33545	25.2378	0.31196	25.04925	0.2556	56.6	7	0.83
Q96I24	Far upstream element-binding protein 3	FUBP3	0.018912	0.2288	19.18425	0.18212	19.52	0.15408	24.7	9	0.81
P18206	Vinculin	VCL	0.019685	− 0.57615	22.07985	0.84436	21.81165	0.52692	64.2	60	0.83
Q96BW5	Phosphotriesterase-related protein	PTER	0.020487	0.35515	23.0556	0.21492	22.8575	0.29673	24.4	6	0.82
P02775	Platelet basic protein; connective tissue-activating peptide III; TC-2; connective tissue-activating peptide III(1-81); beta-thromboglobulin; neutrophil-activating peptide 2(74); neutrophil-activating peptide 2(73); neutrophil-activating peptide 2; TC-1; Neutrophil-activating peptide 2(1–66); neutrophil-activating peptide 2(1–63)	PPBP	0.022319	− 1.4945	21.6995	1.19479	21.55895	1.23096	38.3	5	0.81
P21333	Filamin-A	FLNA	0.023825	− 0.23365	21.21755	0.43258	22.3359	0.24022	71.6	137	0.81
Q01469; A8MUU1	Fatty acid-binding protein, epidermal	FABP5	0.024356	− 0.5329	26.41625	0.73245	26.33375	0.55502	76.3	11	0.83
O94903	Proline synthase co-transcribed bacterial homolog protein	PROSC	0.024792	0.27275	24.465	0.13221	24.3072	0.20226	37.8	8	0.8
P21291	Cysteine and glycine-rich protein 1	CSRP1	0.026425	− 0.2011	25.4829	0.36999	25.34655	0.16603	64.2	8	0.8
P53041	Serine/threonine-protein phosphatase 5	PPP5C	0.028586	0.2748	23.5436	0.14823	23.3623	0.29589	22.8	8	0.84
Q8WUM0	Nuclear pore complex protein Nup133	NUP133	0.030136	0.272	21.15755	0.26096	22.01285	0.19541	18.3	12	0.81
P09525	Annexin A4	ANXA4	0.032901	− 0.25805	26.25495	0.30546	26.6508	0.2431	47.6	13	0.82
Q04826	HLA class I histocompatibility antigen, B-40 alpha chain	HLA-B	0.033546	− 1.0305	21.98915	0.72834	21.76085	0.84434	44.5	13	0.81
O43704	Sulfotransferase family cytosolic 1B member 1	SULT1B1	0.035541	0.4495	26.00495	0.2866	26.19745	0.42792	39.2	9	0.82

The table displays proteins (n = 90) that are differentially expressed in CD4^+^ T cells from MS patients compared to healthy controls (HC). The proteins are extracted from Additional file 1: Table S1 and selected by fulfilling at least two of the three criteria: p-value (p < 0.01), area under the curve (AUC) (AUC > 0.8) and log fold-change (FC) > [0.2] between samples from MS patients and healthy controls. The log2-fold changes in MS versus HC are based on normalized values. Accession number, protein identity and gene names are indicated for each protein, in addition to median log2-transformed protein abundances with standard variation (SD) for each group, the percentage of sequence coverage (% seq cov) and number of peptides (# pep) identified for each protein

**Table 3:** Top-hit list of differentially expressed proteins in CD8^+^ T cells from MS patients compared healthy controls

Accession	Protein identity	Gene names	p-value	FC MS versus HC (log2)	Median intesity MS (log2)	MS SD	Median intensity HC (log2)	HC SD	% seq cov	# pep	AUC
P36915	Guanine nucleotide-binding protein-like 1	GNL1	0.000363	0.3823	22.9373	0.13239	22.555	0.19548	22.6	9	0.9
P57764	Gasdermin-D	GSDMD	0.0004	− 0.247	23.0081	0.09966	23.2551	0.13969	27.9	8	0.91
Q15027	Arf-GAP with coiled-coil, ANK repeat and PH domain-containing protein 1	ACAP1	0.000818	0.3588	25.3317	0.13259	24.9729	0.21057	44.7	22	0.89
Q14240	Eukaryotic initiation factor 4A-II; eukaryotic initiation factor 4A-II, N-terminally processed	EIF4A2	0.001679	0.2287	25.7838	0.1338	25.5551	0.31408	75.4	23	0.92
Q9GZP4	PITH domain-containing protein 1	PITHD1	0.001791	0.1974	22.8647	0.13746	22.3619	0.14509	47.9	8	0.91
P10155	60 kDa SS-A/Ro ribonucleoprotein	TROVE2	0.001865	0.1765	25.7638	0.08234	25.9917	0.13787	37.4	16	0.87
P14174	Macrophage migration inhibitory factor	MIF	0.002217	0.3238	23.462	0.21611	23.2646	0.28198	36.5	4	0.88
Q96ST3	Paired amphipathic helix protein Sin3a	SIN3A	0.002395	0.2302	25.1124	0.11111	24.9359	0.14227	14.2	13	0.85
P06703	Protein S100-A6	S100A6	0.002446	− 0.7296	26.8304	0.62637	26.5066	0.61209	52.2	4	0.9
P51452	Dual specificity protein phosphatase 3	DUSP3	0.002706	− 0.5138	23.1048	0.19565	22.8746	0.4382	29.2	4	0.88
O75431	Metaxin-2	MTX2	0.002927	0.293	25.2256	0.21249	25.9552	0.11457	33.8	5	0.82
Q8TBC4	NEDD8-activating enzyme E1 catalytic subunit	UBA3	0.002937	0.1439	24.5971	0.10266	24.3764	0.1286	51.6	13	0.85
P30405; Q6BAA4	Peptidyl-prolyl cis–trans isomerase F, mitochondrial	PPIF	0.003314	− 1.0344	21.1705	0.33533	21.6843	0.63539	40.1	8	0.84
P21953	2-oxoisovalerate dehydrogenase subunit beta, mitochondrial	BCKDHB	0.003651	0.2586	22.4515	0.21161	22.1585	0.24451	15.3	4	0.83
Q8TCD5	5(3)-deoxyribonucleotidase, cytosolic type	NT5C	0.003791	0.3211	24.2434	0.14217	24.0995	0.45523	54.2	7	0.86
P57737	Coronin-7	CORO7	0.004431	0.147	23.7522	0.148	23.3999	0.0888	49.4	28	0.86
O94925	Glutaminase kidney isoform, mitochondrial	GLS	0.0047	0.1506	22.33535	0.10161	22.9562	0.13926	45	22	0.85
Q3ZCW2	Galectin-related protein	LGALSL	0.005089	− 2.2604	21.7926	0.93109	22.827	0.91857	61	8	0.86
P63151; Q00005; Q9Y2T4	Serine/threonine-protein phosphatase 2A 55 kDa regulatory subunit B alpha isoform	PPP2R2A	0.005319	0.243	21.8042	0.15983	21.5456	0.24976	48.1	12	0.84
O94826	Mitochondrial import receptor subunit TOM70	TOMM70A	0.005477	0.235	23.0302	0.0878	22.7091	0.2231	32.1	13	0.89
Q13586; Q9P246	Stromal interaction molecule 1	STIM1	0.005533	− 0.2404	26.1487	0.20417	26.0017	0.23545	32.7	16	0.82
P13224	Platelet glycoprotein Ib beta chain	GP1BB	0.005768	− 1.9102	24.998	0.8499	24.8474	1.00102	23.8	5	0.82
O00186	Syntaxin-binding protein 3	STXBP3	0.005812	0.193	22.9285	0.11405	23.296	0.15529	10.5	5	0.87
P20645	Cation-dependent mannose-6-phosphate receptor	M6PR	0.006115	− 0.1934	22.3302	0.22866	23.6924	0.17682	22	4	0.84
Q96RQ3	Methylcrotonoyl-CoA carboxylase subunit alpha, mitochondrial	MCCC1	0.007633	0.5028	21.2898	0.19278	23.5502	0.34081	16.4	7	0.83
P78417	Glutathione S-transferase omega-1	GSTO1	0.007795	− 0.2279	23.6102	0.09562	23.1469	0.19987	59.3	14	0.79
P24666	Low molecular weight phosphotyrosine protein phosphatase	ACP1	0.008395	0.2207	23.6258	0.18286	23.3828	0.19924	72.2	8	0.82
Q9H0R4	Haloacid dehalogenase-like hydrolase domain-containing protein 2	HDHD2	0.008617	0.3523	24.1578	0.21888	23.9228	0.23008	67.2	8	0.84
Q12913	Receptor-type tyrosine-protein phosphatase eta	PTPRJ	0.008808	− 0.62085	24.2463	0.30864	24.4867	0.44153	10.9	11	0.83
P49327	Fatty acid synthase; [acyl-carrier-protein] S-acetyltransferase;[acyl-carrier-protein] S-malonyltransferase; 3-oxoacyl-[acyl-carrier-protein] synthase; 3-oxoacyl-[acyl-carrier-protein] reductase; 3-hydroxyacyl-[acyl-carrier-protein] dehydratase; enoyl-[acyl-carrier-protein] reductase; oleoyl-[acyl-carrier-protein] hydrolase	FASN	0.009384	− 0.3675	23.3745	0.18085	25.2847	0.4045	10.9	18	0.8
P04275	von Willebrand factor; von Willebrand antigen 2	VWF	0.009824	− 1.3622	22.1391	1.21897	21.9461	1.05341	13	25	0.84
P35573	Glycogen debranching enzyme; 4-alpha-glucanotransferase; amylo-alpha-1,6-glucosidase	AGL	0.010066	0.4633	21.956	0.26486	21.7305	0.29541	20.9	22	0.8
Q8TDQ7	Glucosamine-6-phosphate isomerase 2	GNPDA2	0.010164	0.2255	23.3542	0.28146	23.8593	0.21281	59.8	10	0.84
P16615	Sarcoplasmic/endoplasmic reticulum calcium ATPase 2	ATP2A2	0.010248	− 0.5051	24.5083	0.30171	24.3585	0.32708	28.7	21	0.81
Q13555; Q13554	Calcium/calmodulin-dependent protein kinase type II subunit gamma; calcium/calmodulin-dependent protein kinase type II subunit beta	CAMK2G; CAMK2B	0.010445	0.2908	22.9757	0.12761	22.6849	0.25143	24.6	10	0.82
P12931; Q9H3Y6; P42685; P08581; Q04912	Proto-oncogene tyrosine-protein kinase Src	SRC	0.01081	− 1.20015	22.8527	0.73685	24.05285	0.66689	37.5	15	0.81
Q15120	[Pyruvate dehydrogenase (acetyl-transferring)] kinase isozyme 3, mitochondrial	PDK3	0.010829	0.24475	20.7149	0.28722	20.47015	0.26592	12.1	3	0.81
P05556	Integrin beta-1	ITGB1	0.010894	− 0.4327	24.5472	0.41637	24.9799	0.37748	32.8	19	0.81
Q9P0J1	[Pyruvate dehydrogenase [acetyl-transferring]]-phosphatase 1, mitochondrial	PDP1	0.011178	0.3474	22.5526	0.14746	22.2052	0.18283	16.9	7	0.82
P01137	Transforming growth factor beta-1; latency-associated peptide	TGFB1	0.012983	− 0.87595	24.8165	0.44917	24.6229	0.65195	29.5	7	0.81
P14770	Platelet glycoprotein IX	GP9	0.013113	− 1.25275	29.9764	0.73326	29.8298	0.84609	30.5	5	0.82
P05386	60S acidic ribosomal protein P1	RPLP1	0.014256	0.2666	22.157	0.16745	22.4676	0.17334	94.7	5	0.81
Q02083	N-acylethanolamine-hydrolyzing acid amidase; N-acylethanolamine-hydrolyzing acid amidase subunit alpha; N-acylethanolamine-hydrolyzing acid amidase subunit beta	NAAA	0.014286	0.43565	23.0686	0.40847	23.6457	0.49594	27.9	8	0.87
P50148; P29992; O95837	Guanine nucleotide-binding protein G(q) subunit alpha	GNAQ	0.014465	− 0.487	24.8592	0.48364	24.5987	0.51884	30.6	8	0.82
O14828	Secretory carrier-associated membrane protein 3	SCAMP3	0.014503	− 0.2404	22.5342	0.16111	22.3259	0.19252	22.8	5	0.8
P67936; Q2TAC2	Tropomyosin alpha-4 chain	TPM4	0.015754	− 0.598	23.0621	0.52458	22.8134	0.46362	70.6	27	0.81
O14561	Acyl carrier protein, mitochondrial	NDUFAB1	0.015958	0.2347	23.5152	0.23866	23.7086	0.12417	21.2	4	0.82
Q00653	Nuclear factor NF-kappa-B p100 subunit; nuclear factor NF-kappa-B p52 subunit	NFKB2	0.016322	0.322	22.11305	0.2449	22.989	0.28366	15.8	9	0.81
P35244	Replication protein A 14 kDa subunit	RPA3	0.016781	0.2976	23.4742	0.16891	24.72695	0.38956	86.8	7	0.8
O95379	Tumor necrosis factor alpha-induced protein 8	TNFAIP8	0.016869	0.2251	22.0409	0.1305	23.8998	0.38972	40.4	5	0.8
Q9NY12	H/ACA ribonucleoprotein complex subunit 1	GAR1	0.01728	0.2208	27.6053	0.18001	28.7912	0.21825	29	5	0.8
P16109	P-selectin	SELP	0.017721	− 2.0401	24.1549	1.03363	23.9684	1.06785	29	14	0.81
Q96RP9	Elongation factor G, mitochondrial	GFM1	0.020163	0.288	24.2375	0.19021	24.3248	0.22518	13.2	7	0.8
Q96F86	Enhancer of mRNA-decapping protein 3	EDC3	0.02024	0.44525	26.7743	0.34422	26.5077	0.41686	12.4	3	0.82
P08134	Rho-related GTP-binding protein RhoC	RHOC	0.022534	0.3601	23.692	0.17871	23.25635	0.48992	65.8	10	0.81
Q15283	Ras GTPase-activating protein 2	RASA2	0.025799	0.2581	25.2002	0.25399	25.0644	0.3012	14.5	9	0.8
O95866	Protein G6b	G6B	0.025824	− 1.4023	21.8137	0.77427	22.3007	0.97969	23.7	5	0.81
O75874	Isocitrate dehydrogenase [NADP] cytoplasmic	IDH1	0.026221	− 0.3508	21.6884	0.25198	21.9288	0.44714	52.4	17	0.81
P09564	T-cell antigen CD7	CD7	0.027969	0.3583	22.542	0.19792	22.6533	0.35246	16.7	4	0.82
O75439	Mitochondrial-processing peptidase subunit beta	PMPCB	0.031524	0.2241	22.4262	0.21703	24.1114	0.27829	22.1	7	0.83
P24158	Myeloblastin	PRTN3	0.049099	− 0.55295	28.221	0.57502	28.819	0.84918	20.7	4	0.81

The table displays proteins (n = 61) that are differentially expressed in CD8^+^ T cells from MS patients compared to healthy controls (HC). The proteins are extracted from Additional file 2: Table S2 and selected by fulfilling at least two of the three criteria: p-value (p < 0.01), area under the curve (AUC) (AUC > 0.8) and log fold-change (FC) > [0.2] between samples from MS patients and healthy controls. The log2-fold changes in MS versus HC is based on normalized values. Accession number, protein identity and gene names are indicated for each protein, in addition to median log2-transformed protein abundances with standard variation (SD) for each group, the percentage of sequence coverage (% sequence coverage) and number of peptides (# peptides) identified for each protein

**Fig. 2:**
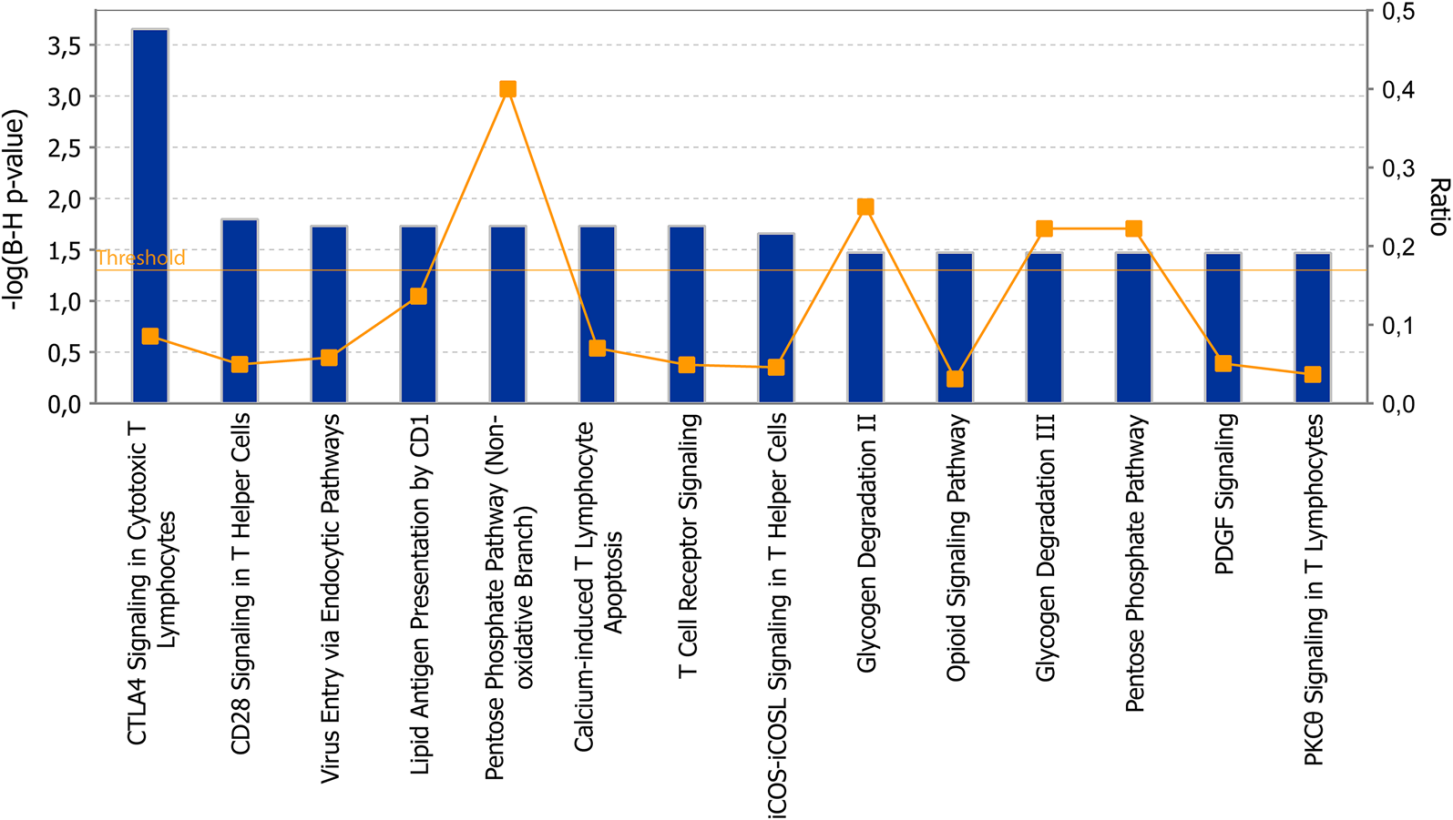
Enriched pathways in CD4^+^ T cells from MS patients. The graph displays the cellular pathways enriched in the proteomic profiles of the top-hit regulated proteins from MS patients as compared with healthy controls in CD4^+^ T cells after correcting for multiple testing (p-value, left axis). The orange line represents the ratio of the number of proteins in the data set of differentially expressed proteins divided by the number of proteins in the reference data set for that specific pathway (right axis)

The ingenuity pathway analyses (IPA) software was used for network analyses of the top-hit proteins (Tables 2, 3) from the CD4^+^ and CD8^+^ T cell data sets separately. After correcting for multiple testing, we identified 14 biological processes in CD4^+^ T cells that were affected by the presence of MS disease (Fig. 2), however, no pathways were significant for CD8^+^ T cells. When performing network analyses of the entire list of 195 differentially expressed proteins (p < 0.05) from CD8^+^ T cells, two pathways were significant after multiple testing, i.e. the sirtuin signaling pathway and the protein kinase A pathway (data not shown). In the CD4^+^ T cell data set, mainly T cell activation pathways, such as CTLA4, CD28, T cell receptor, PKCθ and iCOS-iCOSL signaling and calcium-induced T lymphocyte apoptosis were identified. In addition, general pathways as for instance the pentose phosphate pathway in addition to immune related pathways were represented.

### Hierarchical clustering

**Fig. 3:**
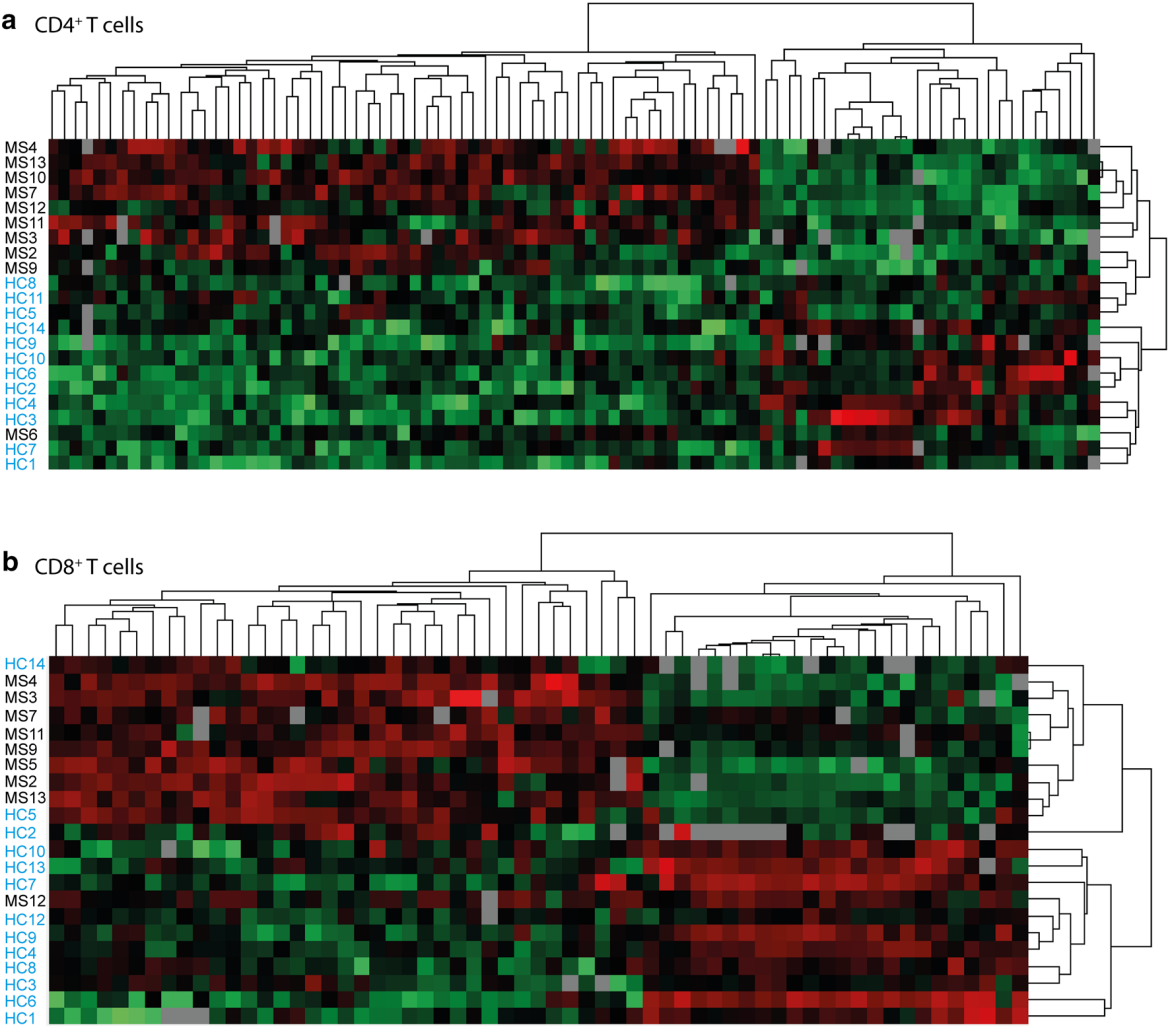
Hierarchical clustering of differentially expressed proteins. The heatmaps show the hierarchical clustering of differentially expressed proteins from the top-hit list fulfilling two out of the three criteria: p < 0.01, AUC > 0.8 and log fold change > [0.2] in **a** CD4^+^ T cells and **b** CD8^+^ T cells from MS patients and healthy control using Perseus. Red: upregulated in MS samples, green: down-regulated in MS samples, grey: missing values. MS (black): samples from MS patients; HC (blue): samples from healthy controls

The normalized intensities of the 90 and 61 proteins in the top-hit list (Tables 2, 3) in CD4^+^ and CD8^+^ T cells from MS patients and healthy controls were used as input to hierarchical clustering in Perseus (Fig. 3). The proteomic profiles for each cell type were divided into two groups consisting mainly of (1) MS and (2) healthy control samples. The differentially expressed proteins are divided into two major groups that are oppositely regulated between MS patients and healthy controls. Using IPA, we did not detect any enrichment for specific biological pathways if we separately analyzed proteins that are either up- or down-regulated in CD8^+^ T cells from MS patients. However, in the proteins that are upregulated in MS CD4^+^ T cells, there is an enrichment for T cell specific activation pathways, in addition to general pathways such as the pentose phosphate and sirtuin pathways. For the proteins that are down-regulated in MS CD4^+^ T cell samples, network analyses in IPA showed enrichment of proteins in integrin signaling and endocytic pathways (data not shown). Of note, we observed three exceptions where two MS patients clustered together with the healthy controls (one for each data set) and one healthy control clustered with MS patients in the CD8^+^ T cell data set.

### Analyses of proteins expressed by MS susceptibility genes

**Fig. 4:**
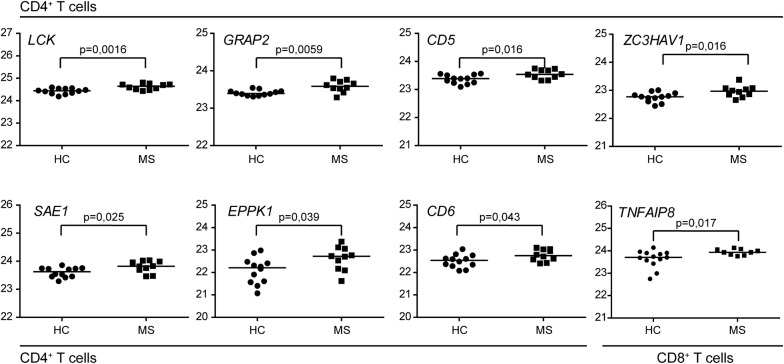
Differential expression of proteins encoded by MS susceptibility genes. The scatter plots represent the log2-transformed protein abundances of proteins expressed from indicated MS susceptibility genes in CD4^+^ T cells and CD8^+^ T cells from MS patients (MS) and healthy controls (HC). Student *t* tests were used to compare the groups as specified in Materials and Methods. The horizontal lines represents the median within the groups

To date more than 200 non-HLA associated MS risk SNPs have been identified by genome-wide approaches [4, 5, 20]. We next selectively analyzed the abundance of proteins expressed from the gene(s) most proximal to these MS-associated SNPs in order to identify proteins with a potential impact on MS disease. For intergenic MS-associated SNPs, we analyzed the abundance of the proteins expressed from the most proximal gene both upstream and downstream of the SNPs. Not all MS susceptibility genes are expressed in T cells, and in our samples, we detected 31 proteins encoded from MS susceptibility genes in CD4^+^ T cells and 37 proteins in CD8^+^ T cells. Of these, eight proteins (seven in CD4^+^ T and one in CD8^+^ T cells) were differentially expressed in samples from MS cases versus healthy controls (Fig. 4).

**Fig. 5:**
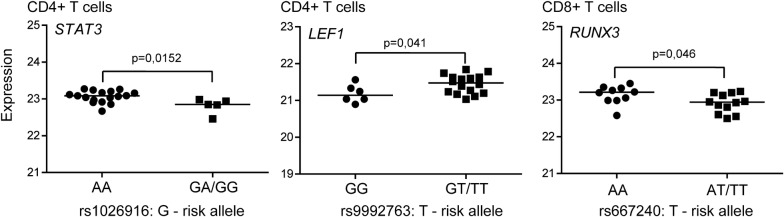
Genotype-dependent expression of proteins encoded by MS susceptibility genes. The scatter plots display the log2-transformed protein abundances of proteins expressed from indicated MS susceptibility genes as function of the MS risk SNP genotype in samples from CD4^+^ T cells (left and middle plot) and CD8^+^ T cells (right plot) from both MS patients and healthy controls sorted for the genotype of indicated MS-susceptibility SNPs. For normalized distributions (LEF1 and RUNX3), Student t-test were performed, otherwise (STAT3), the non-parametric Mann U Whitney test was performed to compare the groups. The horizontal lines represents the median within the groups

To assess the functional link between GWAS-identified risk variants and disease, we evaluated whether there was any correlation between MS risk genotypes and expression of proteins encoded from the most proximal gene(s). For proteins that did not display any difference in abundance in samples from MS cases and healthy controls, i.e. 24 and 36 proteins from CD4^+^ and CD8^+^ T cells, respectively, samples (from both MS patients and healthy controls) were pooled by carriers of the risk allele at each SNP as compared to samples from individuals homozygous for the protective allele for each SNP. We observed a genotype-dependent expression of proteins expressed from the *STAT3* and *LEF1* genes in CD4^+^ T cells and the *RUNX3* gene in CD8^+^ T cells (Fig. 5). However, after multiple testing these correlations did not reach statistical significance.

## Discussion

MS is considered as an autoimmune disorder of the CNS and the pathological immune dysregulation involves an interaction between the innate and adaptive immune system. T cells are thought to be one of the main cellular drivers for disease development, and from genome-wide association screens, a significant enrichment of genetic loci encoding proteins in T-cell specific pathways is observed [5]. Nevertheless functional and epigenomic annotation studies of genetic risk loci suggests that also other cells of the immune system are involved [5, 21, 30]. Proteomic profiling of whole blood or peripheral blood mononuclear cells could contribute to achieve mechanistic insights behind the development of MS pathology. However, such samples are heterogeneous in their cellular composition, so any cell-specific variation may be overshadowed by variation in the proportions of the various cell types. In the current study, we therefore purified CD4^+^ and CD8^+^ T cells and compared their respective proteomic profiles between RRMS patients and healthy controls using liquid chromatography–tandem mass spectrometry. Our study provides evidence for proteomic differences in T cells from RRMS patients compared to healthy controls and identifies three putative pQTLs for proteins encoded by three MS susceptibility genes.

MS is an inflammatory disease that affects the CNS. The cerebrospinal fluid is an obvious fluid to perform proteomic profiling into search for biomarkers of MS, as it reflects ongoing pathological and inflammatory processes in the CNS. However, in the current study, we are examining immune cell subsets, i.e. CD4^+^ and CD8^+^ T cells that enables us to identify proteins and pathways involved in MS development. We are aware of that also other cells of the immune system, including B cells and innate cells such as NK cells and dendritic cells in addition to brain-resident immune cells, i.e. astrocytes [20], have potential impact on MS pathogenesis. However, this study enables us to achieve mechanistic insights into T-cell mediated pathology of MS. Identification of novel proteins and pathways involved in MS pathology could enable progress in the development of new drug targets in order to improve the clinical outcome of MS.

Hierarchical clustering of the differentially expressed proteins from our top-hit list of 90 and 61 proteins from CD4^+^ and CD8^+^ T cells, respectively, divided the samples into two main groups with MS patients and healthy controls. Of note, for each of the cell types, there was one MS patient sample (not the same in the two cell types) clustering with the healthy control group. One of these patients (MS12) has a benign form of MS, and in contrast to all other patients, this patient is currently electively untreated (3 years after inclusion to the study). One healthy control also groups with the MS patients for CD8^+^ T cells; however, whether this control experienced an undetected inflammatory condition or have developed autoimmunity after sample collection giving rise to a proteomic profile similar to MS cases is not known. Even though we have separated immune-cell subsets from the entire pool of immune cells in blood, we acknowledge that these sub-populations can be divided further into different subpopulations such as Th1 and Th2 cells, effector, memory and regulatory T cells. Whether the individuals not clustering with their own group have differences in the proportion of CD4^+^ and CD8^+^ T cell subsets is not known and could potentially affect the proteomic profile achieved. The fold change in protein abundance in T cells from MS patients and healthy controls are modest. However, enrichment in specific pathways (see Fig. 2) suggests that they collectively may have an impact on selected T cell responses. Also, the study is limited by the small sample size, and further studies are needed to validate and verify the biological impact of selected proteins in T cells.

Of the top-ten (based on p-value) differentially expressed proteins in each cell type, only three of them have previously been identified to have a potential role for MS, either through a genetic association, i.e. Lck [20], as a biomarker for MS progression and severity, i.e. macrophage migration inhibitory factor (MIF) [31, 32] or in functional studies, where gasdermin-D (GSDMD) is shown to promote inflammatory demyelination both in human cells and in murine models [33]. Of note, a selection of the top hit proteins in T cells [TAR binding protein (TARDBP), calnexin (CANX) and AP2 associated kinase 1 (AAK1)] have been shown to play important roles for other neurodegenerative disorders such as Alzheimer’s disease, Parkinson’s disease and amyotrophic lateral sclerosis [34–37], suggesting common disease mechanisms across neurodegenerative disorders and highlighting the importance for these proteins also in immune cells.

MS is an inflammatory disease, and therefore it is no surprise that the differentially expressed proteins in CD4^+^ T cells are enriched for pathways related to T cell activation or immune function. Whether these pathways are affected because of the active inflammation that is characteristic for the early phase of RRMS or whether similar changes can be detected prior to disease onset is not known. MS develops in genetic susceptible individuals, and genome-wide screenings have highlighted the importance of genes involved in T cell differentiation, in CD4^+^ T cells in particular [5]. Interestingly, we have identified eight proteins encoded by MS susceptibility genes (*LCK*, *GRAP2*, *CD5*, *ZC3HAV1*, *SAE1*, *EPPK1* and *CD6* in CD4^+^ T cells and *TNFAIP8* in CD8^+^ T cells), which are more abundant in T cells from MS patients compared to healthy controls. This underlines the potential role for these MS susceptibility genes in T cells during MS development prior to disease onset.

Furthermore, correlating MS risk genotype with protein expression from genes proximal to MS risk SNPs, we identified three potential pQTLs, i.e. rs1026916, rs9992731 and rs6672420. Samples from individuals homozygous for the protective allele displayed higher expression of the specified proteins compared to samples from individual being a carrier of the risk allele. Even though these correlations did not reach statistical significance after multiple testing, the data indicate that these SNP-protein pairs are of relevance to study further as the corresponding MS associated SNPs could act as pQTLs. Interestingly, the rs1026916 SNP has previously been shown to act as an eQTL for *STAT3* (at the mRNA level) in skeletal muscle and tibial artery [38]. Rs1026916 lies within a region with moderately high histone H3 acetylation levels, but outside DNAse clusters and transcription factor binding sites [39]. Whether this SNP affects transcription factor binding and thereby regulates transcription remains to be analyzed. Our study further suggests a functional implication of this SNP or a SNP tagged by rs1026916 in T cells. Neither rs6672420 nor rs9992731 are reported to act as an eQTLs [38]. However, the correlation between mRNA and protein copy numbers can vary widely [18, 19] and this study suggests that these SNPs could act as pQTLs in T cells. In contrast to rs9992731 that is not situated in any typical gene-regulatory region, in silico analyses suggests that rs6672420 might affect gene expression, as it is located in a region shown by chromatin immunoprecipitation to be bound by RNA polymerase 2 (POLR2A) and the STAT5A transcription factor [39]. Confirmatory studies in T cells need to be pursued in order to confirm the relationship between genotype at rs6672420, transcription factor occupancy and gene and protein expression of RUNX3. Altogether, the reported pQTLs suggests further exploration of *LEF1*, *STAT3* and *RUNX3* to understand the molecular pathways involved in disease with the ultimate goal to identify new therapeutic targets.

## Conclusion

We show that there is a dysregulation at the protein level in T cells from RRMS patients at an early stage of disease. Pathway analyses, pinpoints to the importance of CD4^+^ T-cell specific activation pathway, which is indicative of an inflammatory condition. By specifically analyzing proteins expressed from MS susceptibility genes, eight proteins were found to be dysregulated in T cells from MS patients. In addition, we identified three novel pQTLs, which might contribute to mechanistically understand the molecular background of MS development and the biology behind three SNPs that have been identified as MS susceptibility gene variants through genome-wide screenings.

## Additional files

**Additional file 1: Table** **S1.** Proteins from CD4^+^ T cells differentially expressed in MS patients and healthy controls. The table displays proteins (n = 228) that are differentially expressed in CD4^+^ T cells from MS patients compared to healthy controls (p < 0.05). For each protein, accession number, protein identity, gene name, log2-fold change in samples from MS versus HC, median log-2 transformed protein abundances with standard variation (SD), the percentage of sequence coverage (% sequence coverage) and number of peptides (# peptides), is given.

**Additional file 2: Table** **S2.** Proteins from CD8^+^ T cells differentially expressed in MS patients and healthy controls. The table displays proteins (n = 195) that are differentially expressed in CD8^+^ T cells from MS patients compared to healthy controls (p < 0.05). For each protein, accession number, protein identity, gene name, log2-fold change in samples from MS versus HC, median log-2 transformed protein abundances with standard variation (SD), the percentage of sequence coverage (% sequence coverage) and number of peptides (# peptides), is given.

## Abbreviations

MS: multiple sclerosis
RRMS: relapsing remitting MS
HC: healthy control
SNP: single nucleotide polymorphism
CNS: central nervous system
EDSS: extended disability status scale
eQTL: expression quantitative trait locus
pQTL: protein quantitative trait locus
PCA: principal component analyses
LFQ: label-free quantification
